# Diversity of susceptible hosts in canine distemper virus infection: a systematic review and data synthesis

**DOI:** 10.1186/s12917-016-0702-z

**Published:** 2016-05-12

**Authors:** Marlen Martinez-Gutierrez, Julian Ruiz-Saenz

**Affiliations:** Grupo de Investigación en Ciencias Animales GRICA, Universidad Cooperativa de Colombia, Calle 30A # 33-51, Bucaramanga, Colombia

**Keywords:** Endangered species, Morbillivirus, Re-emerging virus, Species barrier, Zoonosis

## Abstract

**Background:**

Canine distemper virus (CDV) is the etiological agent of one of the most infectious diseases of domestic dogs, also known as a highly prevalent viral infectious disease of carnivores and posing a conservation threat to endangered species around the world. To get a better panorama of CDV infection in different Orders, a retrospective and documental systematic review of the role of CDV in different non-dog hosts was conducted. The bibliographical data were collected from MedLine/PubMed and Scopus databases. Data related to Order, Family, Genus and Species of the infected animals, the presence or absence of clinical signs, mortality, serological, molecular or antigenic confirmation of CDV infection, geographic location, were collected and summarized.

**Results:**

Two hundred seventeen scientific articles were considered eligible which includes reports of serological evaluation, and antigenic or genomic confirmation of CDV infection in non-dog hosts. CDV infects naturally and experimentally different members of the Orders Carnivora (in 12 Families), Rodentia (four Families), Primates (two Families), Artiodactyla (three Families) and Proboscidea (one Family). The Order Carnivora (excluding domestic dogs) accounts for the vast majority (87.5 %) of the records. Clinical disease associated with CDV infection was reported in 51.8 % of the records and serological evidence of CDV infection in apparently healthy animals was found in 49.5 % of the records. High mortality rate was showed in some of the recorded infections in Orders different to Carnivora. In non-dog hosts, CDV has been reported all continents with the exception of Australasia and in 43 different countries.

**Conclusions:**

The results of this systematic review demonstrate that CDV is able to infect a very wide range of host species from many different Orders and emphasizes the potential threat of infection for endangered wild species as well as raising concerns about potential zoonotic threats following the cessation of large-scale measles vaccination campaigns in the human population.

**Electronic supplementary material:**

The online version of this article (doi:10.1186/s12917-016-0702-z) contains supplementary material, which is available to authorized users.

## Background

Canine distemper virus (CDV) is the etiological agent of one the most infectious diseases of domestic dogs (*Canis familiaris*). It was first isolated by Carré in 1905 [1] and still remains one of the main problems for veterinarians and owners due to the high morbidity and mortality rates [2]. CDV belongs to the *Paramyxoviridae* family, genus Morbillivirus, that includes viruses with epidemiological relevance for human and animal populations such as measles virus, phocine distemper virus, peste des petits ruminants virus, and the eradicated rinderpest virus [3].

CDV is also known as a highly prevalent viral infectious disease of carnivores, posing a conservation threat to endangered species around the world [4–6]. Even recently, it was proposed to rename the virus “Carnivore Distemper Virus” due to its constant threat for different carnivorous species and to commonly documented outbreaks in non-dog carnivores [7].

In domestic dogs, the major route of CDV transmission is through aerosols from respiratory exudates containing the virus, although other body excretions and secretions (e.g., urine) can be infectious. CDV is highly contagious, and viral shedding may follow infection for 60–90 days [3]. However, the question of how wild animals are exposed to CDV remains to be fully addressed and the role of domestic dogs and wild carnivores in the maintenance and transmission of CDV is still not fully understood [8, 9]. It has been shown that CDV can circulate in wildlife even when levels of infection are extremely low and asynchronous to infections found in domestic dogs, supporting the hypothesis that CDV infection is likely to persist in other wildlife species within complex reservoir systems [10].

CDV has also been found in naturally infected non-canine hosts [11] and recent dramatic outbreaks in breeding colonies of rhesus macaques (*Macaca mulatta*) and cynomolgus macaques (*Macaca fascicularis*), has drawn attention of a possible zoonosis in the post-measles eradication times [12].

In an attempt to consolidate the full list of hosts for CDV, a systematic literature review and data assembly was performed to identify relevant studies published in databases of all possible non-dog hosts for CDV (reported infections in species that are not domestic dogs). The search includes published papers reporting serological evaluations, clinical cases, antigenic/genomic confirmation and experimental infections of CDV in non-dog hosts. The aim was to obtain the most comprehensive information available to determine the range of host species susceptible to natural or experimental infection with CDV.

## Methods

### Data collection

This is a retrospective and documental study conducted with printed and Epub ahead of print of available online articles published from May 1964 to September, 2014, following the recommendations made by the Preferred Reporting Items for Systematic Reviews and Meta-Analyses (PRISMA) group and the new PRISMA-P2015 guidelines [13, 14]. Given that these recommendations were originally developed to evaluate healthcare interventions, components of this statement have been customized to fit the scope of the current systematic review as has been done by others [15]. The bibliographic data were collected from the databases of the National Library of Medicine on the web MedLine/PubMed® (http://www.ncbi.nlm.nih.gov/Pubmed) and Scopus® database from Elsevier (http://www.scopus.com/). An Excel® database was constructed with the references of the selected papers, country of report, common animal name and animal species reported infected by CDV. The Family and Order of the CDV infected animals were included according to the Handbook of the Mammals of the World, 2009 [16]. Notes, letters, editorials, news and meeting abstracts were excluded. The data set supporting the results of this article is included as Additional file 1: Table S1 in the web site of the journal.

All reports of serological evaluation, antigenic or genomic confirmation of CDV infection in non-dog hosts were included. Active infections were recorded in cases were the presence of virus, genome or antigen were confirmed (by virus isolation, rt-PCR and/or immunohistochemistry); otherwise, prior infections were recorded based on the presence of antibodies to CDV measured by the virus neutralization test or ELISA test. Only confirmed cases were included; by this, papers reporting clinical signs only were not included unless they also had some antigenic confirmation or histopathology report of findings compatible with CDV infection.

### Search Strategy

For each search in the PubMed and SCOPUS databases, a sentence consisting of different parts joined together by the so-called Boolean operators, i.e., AND, OR, and NOT was used in the search field. The search strategy was not limited by study design or language and no limits to specific years were used. Manual detection was used to exclude publication types, such as notes, letters, editorials, news and meeting abstracts. The words “Canine Distemper Virus” were selected as “MeSH Terms”. As an example of the main search the following text was used: "distemper virus, canine"[MeSH Terms] OR (“distemper”[All Fields] AND “virus”[All Fields] AND “canine”[All Fields]) OR “canine distemper virus”[All Fields] OR (“canine”[All Fields] AND “distemper”[All Fields] AND “virus”[All Fields]. The data was double-checked for all articles included in the qualitative synthesis.

The selection of studies for inclusion was made using a two-step approach. During the first step, researchers selected articles from the search results based on titles and abstracts, excluding those deemed irrelevant to the topic (all those that include dogs as hosts and papers reporting serological evaluation of wildlife CDV vaccination). Disagreements were resolved through mutual consent. The bibliographies of the articles reviewed were scanned for additional older highly relevant literature that could not be indexed in the selected databases. Studies relevant to the research questions were assessed in the second step using the full text, including those studies for which inclusion was uncertain on the basis of title or abstract screen. All articles retained after the first step went through a full-text review performed independently by the two reviewers.

### Statistical analysis

Descriptive statistics were used in all cases to evaluate the results (e.g. the frequency in count and percentage); to measure the trend in publication of CDV infections in different orders throughout the years we used the linear regression (R^2^) as a measure of linear association with a *p*-value of less than 0.05. Statistical analyses were performed using GraphPad Prism® 6 for Windows®.

## Results

**Fig. 1:**
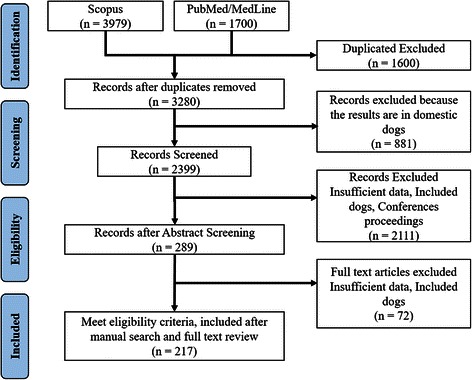
Summary of study search and inclusion (PRISMA flow diagram)

The PRISMA flow chart depicting the process for the systematic literature search and selection of the studies is shown in Fig. 1. Relevant records were assembled from May 1964 to September 2014. Due to the impossibility to eliminate the word “Canine” from the search strategy, the initial search yielded 5679 records (3979 from Scopus® and 1700 from MedLine/Pubmed®).

After the two step screening, 217 papers were deemed eligible that included reports of serological evaluation, antigenic or genomic confirmation of CDV infection in non-dog hosts. Also, the presence or absence of clinical signs were recorded. It is important to note that a single paper can include different records of CDV infected animal Species and/or Orders. A total of 272 different records of infected animals was found (See Additional file 1: Table S1).

As mentioned above, papers including serological and/or immunological evaluation of CDV vaccines in wildlife were excluded. As seen in Table 1, the Order *Carnivora* was the main host reported (87.5 % of the records) and within it, the *Canidae* family was the most commonly reported. Natural infection in twelve of the sixteen families within this Order has been reported [16]. It is important to note that CDV infection among members of the *Felidae* family has become the second most common infection reported in the papers evaluated, even with more reports than among members of the *Mustelidae* and the *Procyonidae* families (Table 1). Although other Orders account for only 12.5 % (36 records) of the infections, it is important to indicate that there are important and endangered species within those Orders, such as Primates, Artiodactyla and Proboscidea.

**Table 1:** Orders, Families and Species that CDV can infect naturally and/or experimentally

Order	Family	Number of records (% of total)	Number of studies reporting clinical signs in infected animals (%)	Species
Carnivora	*Canidae*	82 (30.0)	27/82 (32.9)	*Canis adustus, Canis aureus, Canis latrans, Canis lupus, Canis lupus baileyi, Canis lupus dingo, Canis lupus signatus, Canis mesomelas, Cerdocyon thous, Chrysocyon brachyurus, Lycalopex culpaeus, Lycalopex griseus, Lycalopex gymnocercus, Lycalopex vetulus, Lycaon pictus, Nyctereutes procyonoides, Nyctereutes procyonoides viverrinus, Urocyon littoralis catalinae, Vulpes chama, Vulpes lagopus, Vulpes macrotis mutica, Vulpes velox, Vulpes vulpes, Vulpes zerda*
	*Felidae*	49 (17.9)	24/49 (48.9)	*Acinonyx jubatus, Felis lynx, Felis silvestris, Felis silvestris catus, Leopardus geoffroyi, Leopardus pardalis, Lynx Canadensis, Lynx lynx, Lynx pardinus, Lynx rufus, Panthera leo, Panthera leo persica, Panthera onca, Panthera pardus, Panthera pardus japonensis, Panthera tigris, Panthera tigris altaica, Panthera tigris tigris, Panthera uncial, Puma concolor*
	*Mustelidae*	43 (15.7)	23/43 (67.4)	*Enhydra lutris kenyoni, Galictis vittata, Galictis cuja, Gulo gulo, Lontra Canadensis, Martes foina, Martes martes, Martes melampus, Martes pennanti, Meles meles, Melogale moschata subauantiaca, Mustela erminea, Mustela itatsi sibirica, Mustela lutreola, Mustela nigripes, Mustela nivalis, Mustela putorius, Mustela putorius furo, Mustela vison, Neovison vison, Taxidea taxus*
	*Procyonidae*	18 (6.5)	9/18 (50.0)	*Nasua nasua, Potos flavus, Procyon lotor, Procyon pygmaeus*
	*Ursidae*	13 (4.7)	2/13 (15.3)	*Ailuropoda melanoleuca, Ursus americanus, Ursus americanus floridanus, Ursus arctos, Ursus arctos horribilis, Ursus arctos marsicanus, Ursus maritimus, Ursus thibetanus*
	*Phocidae*	12 (4.4)	8/12 (66.6)	*Halichoerus grypus, Hydrurga leptonyx, Lobodon carcinophagus, Phoca caspica, Phoca sibirica, Phoca vitulina, Pusa caspica*
	*Viverridae*	9 (3.2)	9/9 (100)	*Arctictis binturong, Gennetta gennetta, Paguma larvata, Paradoxurus hermaphroditus, Viverricula indica*
	*Hyaenidae*	6 (2.2)	2/6 (33.3)	*Crocuta crocuta, Hyaena hyaena*
	*Ailuridae*	3 (1.1)	2/3 (66.6)	*Ailurus fulgens*
	*Mephitidae*	2 (0.7)	2/2 (100)	*Mephitis mephitis*
	*Odobenidae*	1 (0.4)	0/1 (0)	*Odobenus rosmarus*
	*Otariidae*	1 (0.4)	1/1 (100)	*Zalophus californianus*
Rodentia	*Muridae*	10 (3.6)	10/10 (100)	*Mus musculus* ^a^
	*Cricetidae*	7 (2.5)	7/7 (100)	*Mesocricetus auratus* ^a^
	*Sciuridae*	1 (0.4)	1/1 (100)	*Marmota caudata*
	*Caviidae*	1 (0.4)	1/1 (100)	*Cavia porcellus* ^a^
Primates	*Cercopithecidae*	7 (2.5)	7/7 (100)	*Macaca fascicularis, Macaca fuscata, Macaca mulatta*
	*Cebidae*	1 (0.4)	1/1 (100)	*Saimiri sciureus* ^a^
Artiodactyla	*Suidae*	2 (0.7)	0/2 (0)	*Sus scrofa domestica* ^a^, *Sus scrofa*
	*Tayassuidae*	2 (0.7)	0/2 (0)	*Tayassu tajacu*
	*Cervidae*	1 (0.4)	0/1 (0)	*Cervus nippon*
Proboscidea	*Elephantidae*	1 (0.4)	0/1 (0)	*Elaphas maximus*
		272 (100)	141/272 (51.8)	

^a^Experimental infections reported

Interestingly, it was found that only 52 % of the records reported clinical disease by CDV. Table 1 show Family by Family the percentage of clinical sings reported on different papers. In these clinical cases, the death of the animals was the most common outcome and CDV was confirmed by immunohistochemical staining of tissues or by rt-PCR. On the other hand, serological reports of CDV infection in healthy animals accounts for almost all the other half of the papers with no clinical signs associated.

### Serological evaluation in members of different Orders infected with CDV

Different serological studies have been performed to evaluate natural CDV infections in non-dog hosts. The most common test used was the virus neutralization test (75.4 %), followed by the Enzyme linked immunosorbent assay (13.8 %), the indirect immunofluorescent assay (7.7 %), the direct neutralizing peroxidase-linked antibody assay (1.5 %) and the dot immunobinding assays (1.5 %).

Most studies (129 records) provided evidence of CDV infection through serological studies that demonstrated seropositivity and that the reported seroprevalence ranged widely across host species within different Orders. Independently of the technique used, the median seroprevalence of all studies/populations on which CDV seropositivity was detected in Carnivora Order was 34,9 %, followed by the Artiodactyla Order with 41.7 % seropositivity and 1.3 % in the Proboscidea Order. Within the Carnivora Order, the *Mustelidae* family, has the highest serological seropositivity with 41.1 %, followed by the *Hyaenidae* with 36.7 %, the *Canidae* family with 35.6 %, the *Phocidae* with 34.8 %, the *Felidae* family with 34,1 %, the *Procyonidae* family with 30.7 %, and the *Ursidae* families with 20.3 %. Table 2 summarizes the minimum and maximum prevalence values reported for different Families and Orders on each study. The full list of prevalence reported for each study can be found in Additional file 1: Table S1.

**Table 2:** Evidence of CDV infection through serological studies. The reported seroprevalence ranged widely across host species within different Orders

Order	Family	^a^Median Seropositivity %	Min % (Sp reported)	Max % (Sp reported)
Carnivora	*Canidae*	35.6	2.0 *Lycalopex culpaeus*	100 *Otocyon megalotis*
	*Felidae*	34.1	3.0 *Puma concolor*	100 *Panthera uncia*
	*Mustelidae*	41.1	5.0 *Mustela vison*	100 *Martes melampus*
	*Procyonidae*	30.7	4.0 *Procyon pygmaeus*	52.0 *Procyon lotor*
	*Ursidae*	20.3	3.0 *Ursus americanus*	100 *Ursus maritimus*
	*Hyaenidae*	36.7	5.0 *Crocuta crocuta*	47.0 *Crocuta crocuta*
	*Phocidae*	34.8	24.0 *Phoca vitulina*	47.5 *Pusa caspica*
	*Mephitidae*	65.5	-	-
	*Ailuridae*	100	-	-
	*Odobenidae*	14	*-*	-
Artiodactyla	*Suidae*	27	-	-
	*Tayassuidae*	58	-	-
	*Cervidae*	40	-	-
Proboscidea	*Elephantidae*	1.3	-	-

^a^Median value of all studies/populations in which CDV seropositivity had been detected in members of the same family. Sp: Specie reported

### Worldwide distribution of CDV in non-dog hosts

**Fig. 2:**
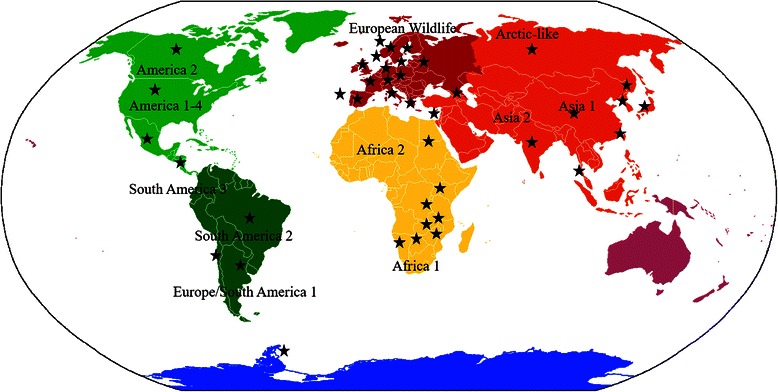
Areas of the world where CDV has been reported in non-dog species. Different CDV lineages are highlighted. Stars denote punctual countries. Map was created using DIVA-GIS software. See text for references

CDV in non-dog hosts has been reported in almost all continents with the exception of Australasia and in 43 countries. Most reports of CDV came from the United States (24.3 %), followed by Japan, Canada and Germany (11.8, 5.5 and 5.1 % respectively). Table 3 presents the full list of countries in which CDV has been reported in non-dog hosts. It is interesting to note that CDV in non-dog hosts was reported in the same continents in which at least one CDV lineage has been identified (Fig. 2).

**Table 3:** Full list of countries in which CDV has been reported in non-dog hosts

Countries	Number of reports
USA	66
Japan	32
Canada	15
Germany	14
Kenya	10
Brazil – China – France	9
Italy – Russia – Spain – Switzerland – United Kingdom – Tanzania	8
Namibia	5
Austria – Norway – Portugal – Thailand	4
Taiwan – South Korea	3
Antarctica – Argentina – Azerbaijan – Bolivia – Chile –Israel – Poland – Zambia - Zimbabwe	2
Botswana – Denmark – Ethiopia – Finland – Guatemala - Greece – India – Luxembourg – Mexico - South Africa – Sudan – Sweden – Uganda	1

### CDV is not a new disease in non-dog hosts

CDV has been reported as a pathogen of dogs for more than one century; however, early records of CDV as an infectious disease for non-dog species was not reported until 1937 during an outbreak among silver jackals (*Vulpes chama*) in a zoo of Johannesburg [17] and then in the Americas: the first case of CDV in the American badger (*Taxidea taxus)* occurred in Colorado in 1942 [18]. The first inter-Order report of CDV occurred in the early 60’s with the development of the hamster model of infection; however due to the non-natural intracerebral inoculation route, this cannot be considered a natural infection. The first report of CDV in clinically healthy wildlife was found during serological evaluation of wolves of Northern Canada, published in 1974; however this report includes samples taken between 1960 and 1964 [19]. Naturally occurring CDV in captive non-human primates (Order Primates) were reported in a Japanese monkey (*Macaca fuscata*) in the late 80´s; however the review shows that experimental infection of macaques could be traced to 1977 [20]. In felids, in 1994, approximately one-third of the lion population in the Serengeti National Park died or disappeared during the best known outbreak of CDV [21]; however, naturally occurring CDV infection in felids was first reported in USA in samples from captive animals infected between 1991 and 1992. It is important to highlight that retrospective tissue evaluations confirmed the presence of CDV antigen in samples from lions and tigers since 1972 in clinical samples belonging to zoos from Switzerland [22].

Taking into account that within the last years, it was claimed that CDV has become an emerging disease for wild animals, we wanted to evaluate if there was a trend in publications of CDV infections in different orders throughout the years. Our results shows that although an exponential tendency is observed (data not shown) we did not find a statistical association between the year of publication and the number of papers reporting CDV infection in different non-dog hosts through the years (*R*^2^ = 0,6113 ; *p* > 0,05). However, it is important to highlight that the lack of statistical association does not reduce the real threat of CDV for wildlife.

## Discussion

CDV is a highly contagious virus that has been tagged as the cause of diseases of strong economic, conservation and public health concern. Although different reviews have been written on CDV infection in wildlife Carnivores [23, 24], this is the first systematic review that goes beyond and investigates the role of CDV in different Orders. Our results show that CDV should be evaluated as a possible cause of disease and extinction risk in different species even outside of the Carnivora Order; as has been proposed for the Tasmanian tiger (*Thylacinus cynocephalus,* Order: Dasyuromorphia*, Family Thylacinidae*) [4, 25, 26].

Importantly though, key role of CDV in Tasmanian tiger extinction could not be confirmed on this systematic review; on the contrary, it was shown that evidence is weak for critical reasons: Evidence for disease is anecdotal and was recorded long after the likely time of the epidemics [25], there is no evidence for CDV infection in any Order belonging to the Infraclass *Marsupialia,* instead evidence showed that there are only reports of CDV in canids in the Australasia continent [27] and finally, recent reports using mathematical modelling has shown that thylacine’s extinction could be explained by interactions between known historical stressors, without involving diseases such as CDV [28].

Phylogenetic studies based on the complete sequence of the hemagglutinin gene (HA) of several CDV strains isolated in distinct geographical locations around the world have revealed a phylogeographic pattern of genetic diversity. According to this pattern, there are fourteen distinct lineages known as America 1 (that includes almost all commercially available vaccines), America 2, America 3, America 4, Arctic-like, Rockborn-like, Asia 1, Asia 2, Africa 1, Africa 2, European Wildlife, Europe/South America 1, South America 2 and South America 3 [29–34], although recently, a new criterion establishes that strains with <2 % divergence at the HA protein level within a single clade should be classified as subgenotypes [35]. According to the present systematic review, CDV in non-dog hosts has been reported in almost all continents and in 43 countries (Fig. 2); there were no recent reports of CDV infection in Australian non-dog hosts, although CDV is a well known dog pathogen in this continent [27] and it has been reported anecdotally that caused several epidemics in dingoes (*Canis lupus dingo*) in central Australia in the 60s and 70s [36]

It is important to highlight that most of CDV infections reported in this systematic review belong to CDV infections in captive wild animals. It is clear that conditions of captivity and the presence of different mammalian species in and around zoos increase the risk of CDV transmission among many animal species [37, 38]. However, the presence of CDV cases in non-dog hosts could be completely unrelated to the status of CDV in free-ranging wildlife in the same country.

Table 2 demonstrates serological evidence for CDV infection in a wide range of families and orders. Seropositivity was expressed as a median value of all studies within a given Family, however the aim of this review was to show the role of species susceptibility to CDV, independently of the serological technique used or whether the animals were free or captive; that is why these results cannot be taken as a reference of worldwide of CDV prevalence in a determined species and region.

Amino acid variations in the CDV HA protein that bind cellular SLAM (signaling lymphocyte activation molecule) are thought to be important in species specificity. In fact, two mutations have been reported in residues 530 (G/E to R/D/N) and 549 (Y to H) that are involved in the inter-species transmission from domestic dogs to non-dog hosts [4]. Although a recent analysis has suggested a bias with respect to the 530 substitutions [39], two recent papers performing molecular phylogeography of CDV has independently shown, that residues 530 and 549 of the CDV HA have been subject to positive selection [32] and that although genetic flow among domestic and wildlife hosts occurred, the domestic dog host appears as the most probable virus reservoir throughout the evolution [40].

Genbank contains as yet too few CDV sequences isolated from naturally infected hosts belonging to other Orders (such as Primates, Artiodactyla or Proboscidea) to allow evaluating the possible role of these amino acid residues in the ability of CDV to infect members of new Orders and Species. To date, analysis of these residues in pathogenic CDV strains from captive and wild felids has demonstrated no correlation between these amino acid residues and pathogenicity in these felids, and additional factors may be more important than the HA protein in determining the infectivity and pathogenicity of CDV strains in various hosts [7]. We believe that a similar evaluation must be performed for CDV, at the genomic and amino acid sequence levels, isolated from different wild species and from different Orders trying to rule out the role of those mutations in interspecies transmission and in virus pathogenesis in new hosts [41, 42]. Although some efforts have been made in this direction [39], we believe that these studies must include larger datasets not only with sequences belonging to the order Carnivora, but also sequences from other orders that have been shown can become infected.

There have been few reports of CDV in domestic cats (*Felis silvestris catus*). Serological evidences (ranging from 4.5 to 23 % prevalence) [43, 44] have shown that some domestic felines can be exposed to CDV and develop antibodies. In vivo, it was demonstrated years ago that CDV can infect domestic cats [45]; in vitro, it has been shown that feline cell lines can be efficiently infected with CDV [46] either expressing canine [47] or feline SLAM [48]. However, there is as yet no report of distemper disease in domestic cats, maybe due to a natural resistance to the disease by controlling viral replication at initial stages of the disease and only suffering a subclinical disease with a slight increase in temperature as have been experimentally reported [45].

Here we show that only 49 % of the reported records of CDV infections in the *Felidae* family presents clinical sign of the disease (Table 1); moreover, the median serological seropositivity for the *Felidae* Family was 34.1 % (ranging from 3 to 100 %). This serological confirmation of CDV in healthy animals means that some animals can acquire the infection, maybe suffer a subclinical and/or asymptomatic disease and remain seropositive for years.

Different outbreaks of CDV has been recorded in the Order Primates [49, 50], the same Order as *Homo sapiens sapiens*. As shown in Table 1, in Primates, all studies reporting evidence of CDV infection also reported clinical signs, independently of the Species involved. In all studies, the reasons for the epidemic remain unclear and the source of the infection is unknown, because there were no dogs or other animals in the farms. One possible source of infection is contact between the monkeys in the farm and local wild monkeys or the spillover of the virus from a stray animal carrying CDV that became adapted to the new host [50, 51].

Such outbreaks in non-human primates have highlighted the hypothesis of a possible zoonosis in the post-measles eradication era. Until now, due to the fact that SLAM and nectin-4 receptors present high homology in their amino acid sequences between monkeys and humans [49], different studies have shown that CDV could potentially become capable of using human cell receptors [52] indicating that CDV infection could be a potential future threat for humans [53], since it appears that so far, zoonotic CDV potential has been controlled by measles vaccination in humans [12].

Antigenic and/or genetic confirmation of CDV outbreaks in wildlife it is an important tool to evaluate the role of CDV as a threat for non-dog host populations, and it is an optimal approach to confirm interspecies viral transmission among wildlife and dogs that could be driven by the urban ecosystem [54]. Although our results show that 51.8 % of studies report clinical signs in such infected animals (Table 1), the mortality rate could be high for some species, as has been reported in some natural and/or experimentally infections [11, 55]. For example, in some families of the Carnivora Order (such as the *Viverridae*, *Mephitidae* and *Otariidae*), the most common outcome of CDV infection reported in the database was the death of infected animals [56–59]. However, these reports were based on case studies each involving a single individual, and to determine the impact of CDV in these important families, population-level studies would be needed [56–59].

This indicates a high risk of extinction for endangered populations as has been stated for the Amur tigers (*Panthera tigris altaica*) [6, 8] and for the Giant pandas (*Ailuropoda melanoleuca*) [38]; in these populations, recent outbreaks of CDV have become an important cofactor in reducing free-ranging wildlife animals and even in a few breeding centers, demonstrating the need to implement mitigation strategies such as vaccination or antivirals to reduce the heavy impact of this infection on those populations [7, 38, 53]

It is important to highlight that periodic serological and molecular evaluations, and an active search for possible wildlife reservoirs of CDV could help prevent outbreaks of diseases in other susceptible wildlife [60], and that domestic dogs, mesocarnivores or peridomestic wild animals (not only carnivores) should be taken into account as possible sources of CDV infections in wildlife [7, 38, 61]. The wide variety of possible hosts for CDV that we were able to find, agrees with recent papers showing that domestic dogs are not the only potential reservoir of CDV. As has been shown in the case of CDV infection in lions in the Serengeti ecosystem, the virus can circulate in lions even when levels of infection are extremely low and asynchronous to infections found in domestic dogs [10], supporting the hypothesis that CDV infection is likely to persist involving the wider domestic dog population beyond the Serengeti ecosystem and other wildlife species.

The highly common presence of CDV seropositive healthy animals (Table 2) is an indication of viral circulation, infection and recovery of animals belonging to different Orders. We believe that those species may act as wildlife reservoirs and that in some cases, some interconnected populations may function as a “metareservoir” consisting of multiple interconnected populations acting as one single revervoir in nature, on which CDV might be maintained through interaction of multiple subpopulations, each experiencing intermittent but non-simultaneous CDV epizootics [62, 63].

The finding of evidence of CDV infection in naturally infected Orders (Tables 1 and 2) such as Artiodactyla (Family Tayassuidae) [64], Rodentia (Family Sciuridae) [65] and Proboscidea (Family Elephantidae) [66] suggests that further serological and molecular CDV evaluation is warranted to investigate patterns of infection, disease risk and sources of infection in both free-ranging and captive populations. For example, the presence of CDV antibodies in Asian elephants (*Elephas maximus*) in Thailand was not associated with the presence of domestic dogs nor with clinical signs of disease [66], although there is currently no evidence that the Asian elephant is a competent host for virus replication or that CDV may cause disease in this endangered species [67].

Serological data have potential value for estimating pathogen force of infection and transmission rates, and have been used in dynamic disease models [68], as demonstrated by studies of the dynamics of cross-species transmission between domestic dogs and lions in the Serengeti ecosystem [10]. A decrease in seroprevalence could also signal a loss of herd immunity which may have value in assessing risks of potential outbreaks and/or need for implementation of preventive measures [10, 69].

Although the Ferret (*Mustela putorius furo*) has served as the most important animal model to study CDV pathogenesis and transmission [70], there have been many CDV models of infection. For instance as can be seen in Table 1, in all orders on which experimental CDV infection have been performed, natural infection has been shown in at least one other Family of the same Order. Hence, although experimental infection is carried out by an unnatural route, these studies demonstrate the potential for CDV to infect members of the Order. For example, experimental infections have been demonstrated in domestic suids (*Sus scrofa domestica*) with serological evidence for endemic infection in wild populations, as has been reported for the collared peccary (*Tayassu tajacu*) [64] Similarly, experimental infections have been demonstrated in several species of the Order Rodentia, and molecular detection of CDV with clinical signs of disease recorded in captive marmot populations (*Marmota caudate*) [65].

Finally, it is well known that jumps in species barriers is how a pathogen can find novel hosts; it requires a complex process of invasion of and adaptation to a new niche [71]. The new host may present challenges at the level of virus entry into cells, virus replication or transmission from the host to finally cause diseases, outbreaks and pandemics [72]. CDV is not just able to infect multiples species; indeed, the present analysis shows that CDV can infect five different Orders. As far as is known, only a few critically emerging and re-emerging viruses such as the Equid herpesvirus 9 causing fatal encephalitis [73], the West Nile Virus [74] or the Influenza A virus [75] have been reported to infect five or more different Orders.

Although no statistical association was found between the number of articles reporting CDV infection in non-dog host and year of publication, CDV is now being reported more frequently in wild animal populations [8, 10]. However, it cannot be ruled out that this increasing tendency could be biased by the increasing number of international journals indexed in the last years in those databases and the increasing number of papers published in this research area [76, 77].

### Strengths and limitations of this review

The major strengths of this systematic review are the characteristics of the selected studies and the wide time window examined. Furthermore, this systematic review allowed us to propose a hypothesis that will be useful to implement future epidemiological research trying to understand the role of mesocarnivores and peridomestic wild animals in the CDV transmission cycle and the role of point mutations in the CDV HA protein in cross species CDV infection.

All of the selected studies were relevant to CDV in non-dog hosts; however, despite of the strength of the search, due to the impossibility to discard the word “canine” from the search keywords, there are some relevant information that could be dismissed because the main focus of the abstract and the title of the paper concerned dogs. Other important limitations were the role of serological techniques used in the different papers reviewed; over the years, authors have used different serological techniques to assess the presence of anti-CDV antibodies in different animal species. Therefore it was not possible to consolidate data related to specific antibody titers, sensitivity or specificity of the techniques used in the papers evaluated.

It is important to note that although the virus neutralization test was shown to be the standard assay used to detect CDV-specific antibodies in serum of different species (75.4 % of records), it requires a large volume of serum, special facilities (biosafety level-2) and a high level of technical skill for the correct manipulation of live virus and cells. Currently, the ELISA test has become a very useful technique to detect anti-CDV antibodies; besides, it does not use live CDV, it requires only a small amount of sample, the procedure to detect antibodies against CDV is simple in many animal species and in many different part of the world that do not have special biosafety laboratories. Also, the ELISA test has shown high specificity and sensitivity in comparison to the virus neutralization test by using anti-dog IgG or IgM antibody as a secondary antibody [78] or by using protein A/G as a secondary antibody to detect the CDV antibody in various mammalian species [79].

Furthermore, the exclusion of studies of CDV vaccine evaluations in wildlife is another limitation of this review; however the aim of the systematic review was focused only on non-vaccinated animals in order to highlight the role of CDV as an important virus for different Species/Orders. Future reviews are being prepared to clarify the role and efficiency of CDV vaccination in wild, captive and free ranging animals.

Finally, database selection could be introducing some bias in the selection of the studies [80]; however, such bias is accepted due to the importance and worldwide relevance of the selected databases.

## Conclusion

The present data support the hypothesis that CDV presents a high potential as emerging virus for wild species as for domestic animals. Also CDV is a worldwide threat for a wider range of host species than is generally recognized and that there is a chance for a possible adaptation of CDV to humans after cessation of measles vaccination as has been suggested by others [12]. The present data also highlights the possible role of CDV as a potential disease threat to a wide range of endangered wild free-ranging and captive species.

## Additional file

Additional file 1: Table S1. Full database of CDV infection in non-dog hosts. (XLSX 31 kb)

## Abbreviations

CDV: canine distemper virus
ELISA: enzyme-linked immuno sorbent assay
HA: hemagglutinin gene
PRISMA: preferred reporting items for systematic reviews and meta-analyses
rt-PCR: reverse transcription polymerase chain reaction
SLAM: signaling lymphocyte activation molecule
